# A longitudinal evaluation of psychosocial changes throughout orthognathic surgery

**DOI:** 10.1371/journal.pone.0203883

**Published:** 2018-09-12

**Authors:** Ka Shing Suen, Yihuan Lai, Samuel M. Y. Ho, Lim Kwong Cheung, Wing Shan Choi

**Affiliations:** 1 Oral and Maxillofacial Surgery, Faculty of Dentistry, The University of Hong Kong, Hong Kong SAR, China; 2 Psychology Laboratory, Department of Social and Behavioural Sciences, City University of Hong Kong, Hong Kong SAR, China; Universidade Federal Fluminense, BRAZIL

## Abstract

**Background:**

Jaw correction surgery can cause significant psychosocial impacts on patients. This prospective study investigated the longitudinal changes of psychosocial characteristics of patients with dentofacial deformities after jaw correction surgery and the factors that predict the psychological resilience in Hong Kong Chinese undergoing jaw correction surgery.

**Methods:**

A longitudinal cohort study was conducted on 92 Hong Kong Chinese patients (32 males, 60 females; mean age = 24.75 ± 5.65 years), who had jaw correction surgery as treatment for their dentofacial deformities, from 1^st^ June 2011 to 30^th^ June 2015. Self-completed psychological inventories including Brief Symptom Inventory, Life Orientation Test, and the Adult Trait Hope Scale were used to measure distress, optimism, and hope levels respectively. Patients completed the inventories in five time points: the surgical consent signing day (usually two to three months before the surgery) (T1); one day before operation (T2), first to second post-operative week (T3), third post-operative month (T4) and sixth post-operative month (T5).

**Results:**

Latent class growth analysis revealed two outcome trajectory classes: a resilience trajectory (n = 45, 48.9%) and a chronic dysfunction trajectory (n = 14, 15.2%). Another 33 (35.9%) showed erratic trajectory patterns that would not be classified into any categories. The psychological distress levels of patients in the resilience trajectory group, on average, were below the clinical threshold of the Brief Symptom Inventory at all time points. However, the opposite result was obtained for patients in the chronic dysfunctional group. Patients exhibiting a resilience trajectory pattern, when compared to those showing a chronic dysfunction pattern, had higher optimism (t(57) = 3.69, p < .0001) and hope (t(57) = 2.46, p < .05) levels at T1. Logistic regression analyses were conducted to compare the relative power of optimism and hope levels at T1 to predict resilience or chronic dysfunctional group membership. A test of the full model against a constant only model was statistically significant (χ^2^(2) = 24.096, p < .01). Preoperative baseline optimism (B = —.276, p < .05) but not hope (B = —.25, ns) was a significant variable to classify the outcome trajectories for psychological distress.

**Conclusions:**

Most patients were resilient to dentofacial deformities jaw correction surgery. About 15% exhibited a chronic distress pattern. An optimistic view about the surgery may enhance resilience. Pre-surgical counselling or educational sessions to facilitate a realistic positive outlook about the operation would be beneficial.

## Introduction

### Dentofacial deformities

Human face can be regarded as the unique centre of attention during social interaction. People with dentofacial disfigurement are commonly presented with disharmonised facial skeletons and malocclusion. The deformity may be caused by over-development (hyperplasia) or under-development (hypoplasia) of the facial skeletons. They can involve single jaw, both jaws and even multiple craniofacial structures. The misaligned teeth can contribute to problems with speech and mastication.

### Treatments for correcting dentofacial deformities

Orthognathic surgery for correction of dentofacial deformities has been developed for over 50 years and it is considered as a mature technique performed by maxillofacial surgeons worldwide. It aims to reposition the jaws into harmonised anatomical positions so that normal facial profile and occlusion can be achieved. Most of the patients with dentofacial deformities usually undergo a combined orthodontic treatment and orthognathic surgery to correct their deformities and improve occlusion. Orthodontic treatment is carried out to align the crooked teeth as a pre-surgical preparation for the jaw correction surgery [1]. Skeletal growth of the patient is assessed and the surgery is usually planned after the skeletal growth is stabilised at around age 17 to 18. Post-surgical orthodontic treatment usually follows so as to optimise the interdigitation of teeth and harmonise the dental arches.

### Prototypical psychological outcome trajectories after a stressful event

**Fig 1:**
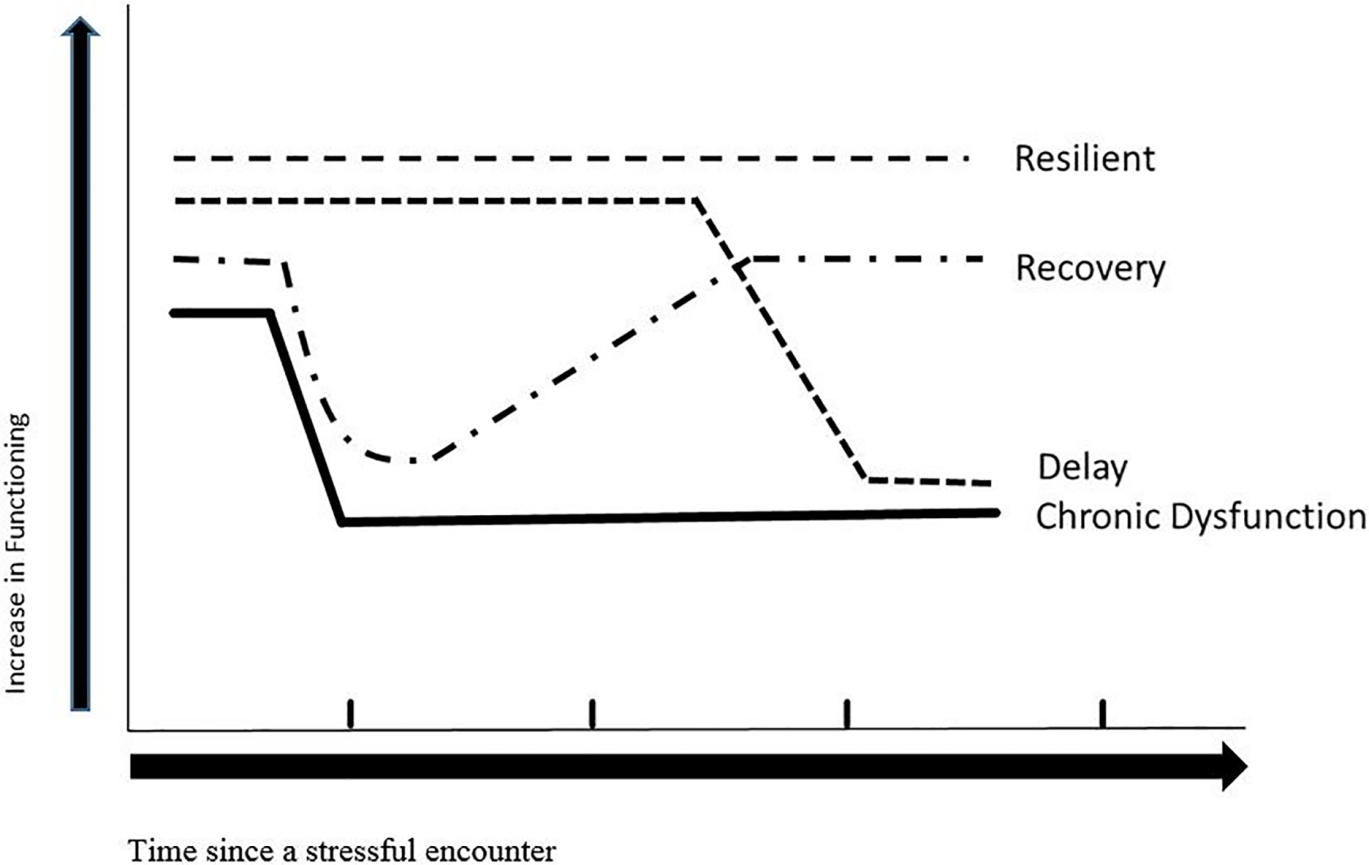
Prototypical outcome trajectories after a stressful encounter.

Orthognathic surgery can be considered as a potentially traumatic event, which can cause post-operative stress. It has been reported that there are different individual psychological responses to traumatic event over time [2] and four prototypical patterns or trajectory outcomes that represent most people’s long-term psychological responses after a potentially traumatic event were identified [3–6]. The four trajectories include resilience, chronic dysfunction, recovery and delayed reaction [6]. Resilience represents individuals who maintain a stable and healthy level of psychological and physical functioning after a traumatic event [3, 5–7]. Chronic dysfunction category describes the individuals with a sustained pattern of elevated symptoms and distress. Recovery represents individuals who initially show elevations in symptoms and distress, followed by a gradual reduction and return to the population norm. The delayed reaction group are individuals who initially show moderate symptom level (sub-threshold) after a potentially traumatic event, followed by a gradual increase to above-threshold elevation over time [6, 8, 9]. Fig 1 depicts these four prototypical psychological outcome trajectories.

### Hope and optimism in traumatic surgery

Hope is a goal-oriented positive motivational state consisting of the agency and pathways components [10, 11]. The agency component is the willpower used to initiate and sustain the movement toward achieving a goal whereas the pathways component refers to the perceived ability in generating successful strategies for goal attainment [12]. Optimism is generally conceptualized as a stable tendency to believe that good rather than bad things will happen [13]. Although both hope and optimism are adaptive cognition, it has been shown that they are separated constructs and may generate desirable outcomes via different mechanisms [14, 15]. In a retrospective cross-sectional study among patients with oral cavity cancer, it has been shown that both hope and optimism were negatively correlated with depression and anxiety symptoms [16] and positively related to self-perceived positive changes [17]. Regarding outcome trajectories, a longitudinal study among individuals going through hereditary colon cancer screening has shown that baseline hope level was a significant predictor of resilience outcome trajectory [18]. In other words, higher hope individuals, when compared to their lower hope counterparts, has higher tendency to exhibit a resilience outcome trajectory during hereditary colon cancer screening.

### The present study

This prospective longitudinal study aimed to investigate the longitudinal psychosocial changes throughout the treatment pre-operatively to six months post-operatively. It also investigated whether preoperative dispositional hope and optimism levels of patients with dentofacial deformities can predict prototypical psychological outcome trajectories after jaw correction surgery. It is hypothesised that patients with high dispositional hope and optimism would exhibit a higher tendency to show a resilience outcome trajectory pattern than those with a lower dispositional hope and optimism level.

The current study fills in the literature gap since, to the best of our knowledge, no previous study has yet investigated the prototypical outcome trajectories following jaw corrective surgery in patients with dentofacial deformities.

## Materials and methods

### Patients and procedures

Patients with dentofacial deformities were recruited from the Discipline of Oral and Maxillofacial Surgery, Faculty of Dentistry, University of Hong Kong between 1^st^ June 2011 and 30^th^ June 2015. Eligible participants were Chinese and aged 18 or above, with significant dentofacial deformity and they were indicated for combined orthodontic and orthognathic surgery or distraction osteogenesis. Patients with cleft or craniofacial syndromes, previous maxillofacial surgery in the last 6 months and history of psychological or psychiatric disorders were excluded. The study was approved by the University of Hong Kong / Hospital Authority Institutional Review Board (HKU/HA HKW IRB ref no.: UW 11–268) and all patients involved provided their written informed consent.

A repeated measures longitudinal design was adopted. All patients underwent orthognathic surgery under general anaesthesia in Queen Mary Hospital of Hong Kong. After written informed consent, the patients were asked to complete a set of questionnaires at five time-points: the surgical consent signing day (T1, usually two to three months before the surgery); one day before surgery (T2), first to second post-operative week (T3), third post-operative month (T4) and sixth post-operative month (T5).

### Measures

#### Brief symptom inventory

The Brief Symptom Inventory (BSI) is a 53-item self-completed inventory designed to measure psychological symptoms among individuals [19]. A BSI total score was obtained by summing all the item scores. The BSI Global severity index (GSI), which is the mean of all 53 items, was used to measure global psychological distress in the present study. Derogatis [20] stated that the GSI is a highly sensitive measure with T-scores greater than or equal to 63 suggesting significant emotional turmoil. Cronbach’s reliability alphas of the BSI total score were excellent and around .97 at all time points.

#### Adult Trait Hope Scale

The Trait Hope Scale (Hope) [21] is a 12-item measure of a respondent’s level of hope, consisting of the 4-item Agency subscale and the 4-item Pathways subscale. The remaining 4 items are fillers. Responses to each item are made on an 8-point Likert scale ranging from 1(definitely false) to 8 (definitely true). A Hope total score is calculated by summing the agency and pathways subscale scores. The Chinese Adult Trait Hope Scale has been developed and used extensively in previous research among dental patients [16, 17]. Cronbach’s alphas according to the present sample ranged from .88 to .94 at the five time points [18].

#### Life Orientation Test—Revised

The Life Orientation Test—Revised (LOT) was constructed by Scheier et al. [22] to measure an individual’s level of optimism versus pessimism. The scale consists of six items. Participants respond to each item using 5-point scale ranging from 1 (strongly disagree) to 5 (strongly agree). The Chinese version of the LOT-Revised was developed by Lai and Yue [23] and has been used in previous dental research [17]. Cronbach’s reliability alphas according to the present sample ranged between .76 and .77 from T1 to T5.

### Statistical analysis

Means and standard deviations of GSI, optimism and hope scores at each time point were described. We used MPlus 7.4 to conduct latent class growth analysis (LCGA) to establish trajectory patterns of the GSI scores from T1 to T5. It was suggested that the number of classes should be determined by a number of factors including both research questions and fit indices [24]. Most of the previous studies have shown four classes of trajectory outcome patterns [4, 5, 18, 25]. Based on these results, we compared one- to four-class unconditional LCGAs (no covariates) in this study first. Bayesian (BIC), sample-size adjusted Bayesian (SSBIC), Aikaike (AIC) information criterion indices, entropy values, the Vuong-Lo-Mendell-Rubin likelihood ration test (VLMR-LRT), the Lo-Mendell-Rubin likelihood ratio test (LMR-LRT) [24], and the bootstrap likelihood ratio test (BLRT) was used to indicate goodness-of-fit. A high entropy value, a low BIC value, a significant LMR-LRT p-value and VLMR-LRT p-value comparing the k and k-1 model are preferred. Optimism and hope was included as covariates and conditional LCGAs were conducted subsequently to examine the degree of correct model specification [26].

Descriptive statistics were provided including distribution of the outcome trajectory patterns. Independent sample t-tests were conducted to examine the differences, if any, between the resilient and chronic dysfunctional outcome trajectories in T1 optimism and hope score. Because we had a categorical outcome variable, binominal logistic regression was used to examine the relative predictive power of hope and optimism in predicting group membership of resilience or chronic dysfunctional outcome trajectories.

## Results

### Subjects

133 patients (45 men and 88 women) with dentofacial deformities were recruited consecutively. 92 subjects consisting of 32 men and 60 women (mean age = 24.75, SD = 5.65), who had completed the BSI-GSI in all five time points were included in this study. Most of the subjects underwent double jaw surgery including Le Fort I osteotomy with segmentalization (64.1%), mandibular anterior subapical osteotomy (58.7%) and / or genioplasty (60.9%) and mandibular ramus osteotomy (91.3%) (Table 1). There were no significant differences in gender distribution (χ^2^(1) = .12, ns), mean age (t(116) = -.003, ns), and mean BSI GSI (t(127) = -1.20, ns) scores between the included and excluded subjects. Power analysis showed that statistical powers ranged from 0.89 to 0.93 were achieved based on a moderate effect size of 0.3 and an alpha equal to 0.05 [27].

**Table 1:** Summary of surgical procedures performed.

Surgical Procedure	Number of subjects
Maxillary surgery	
Le Fort I without segmentalization	26
Le Fort I with segmentalization	59
Simultaneous Le Fort III and Le Fort I	1
No surgery	6
Mandibular surgery	
With ramus osteotomies	84
Without ramus osteotomies	8
With subapical osteotomy	54
Without subapical osteotomy	38
With genioplasty	56
Without genioplasty	36

### Descriptive statistics

The mean (SD) BSI GSI, optimism, and hope scores in each of the time-points are shown in Table 2.

**Table 2:** Mean (SD) of psychological variables in each time-point.

	Surgical consent signing date (T1)	1 day before operation (T2)	1^st^– 2^nd^ post-operative week (T3)	3^rd^ post-operative month (T4)	6^th^ post-operative month (T5)
	Mean (SD)	Mean (SD)	Mean (SD)	Mean (SD)	Mean (SD)
BSI GSI	.59 (.44)	.49 (.42)	.49 (.43)	.47 (.42)	.48 (.45)
Optimism	20.40 (3.93)	21.05 (3.92)	20.73 (3.86)	20.19 (3.86)	20.23 (3.88)
Hope	48.50 (7.16)	47.31 (7.08)	46.87 (8.00)	47.20 (7.83)	47.13 (8.46)

Men and women did not show significant differences in any of the variables at each time point. Age was also not significantly correlated with any psychological variables in all time-points. We aggregated the data for outcome trajectories classification.

### Latent class growth analysis (LCGA)

#### Unconditional model

The primary aim of this study is to examine the distribution of the four outcome trajectories among patients undergone orthognathic surgery. We conducted LCGA (unconditional model) first. The fit indices including AIC, BIC and SSBIC decreased from one- to four-class solutions. The entropy figure increased from two-class to three-class, and decreased from three-class to four-class. Both the VLMR-LRT p-value and the LMR-LRT p-value were very significant in the three-class model, but did not reach significance in the four-class model. Therefore, the three-class trajectory pattern was initially adopted as the best fitting solution.

#### Conditional model

It is proposed that conditional LCGA to include covariate predictors of class membership should be conducted to examine the degree of correct model specification [26]. We used hope and optimism at T1 as covariate predictors in our LCGA (conditional model) based on previous research among patients with oral cavity problems [16, 17]. Table 3 shows that the fit indices including AIC, BIC and SSBIC decreased from one to four-class solutions. Both the VLMR-LRT p-value and the LMR-LRT p-value were very significant in the two-class model, but did not reach significance in the three-class model and four-class model. Furthermore, the entropy value decreased from the two-class model to the four-class model. Accordingly, the two-class trajectory pattern was adopted as the final best fitting model to our data.

**Table 3:** Fit indices for one to four class growth mixture models (conditional with hope and optimism set as covariates).

Fit Indices	Growth mixture mode			
	One class	Two classes	Three classes	Four classes
AIC	489.98	257.87	193.71	120.05
BIC	517.48	297.87	246.21	185.04
SSBIC	482.76	247.37	179.93	102.98
Entropy	-	.95	.94	.92
VLMR-LRT p value	-	.03	.30	.15
LMR-LRT p value	-	.03	.30	.16
BLRT p value	-	< .001	< .001	< .001

**Note**: AIC: Akaike information criterion; BIC: Bayesian information criterion; SSBIC: sample size adjusted Bayesian information criterion; VLMR-LRT: Vuong-Lo-Mendell-Rubin likelihood ratio test; LMR-LRT: Lo-Mendell-Rubin likelihood ratio test; BLRT: bootstrap likelihood ratio test.

According to the two-class model, 45 out of the 92 patients (48.9%) were grouped into Trajectory I and 14 of them (15.2%) belonged to Trajectory II. Another 33 patients (35.9%) showed trajectory patterns that could not be classified into any categories.

### Trajectory of psychological distress in patients with dentofacial deformities after correction surgery

**Fig 2:**
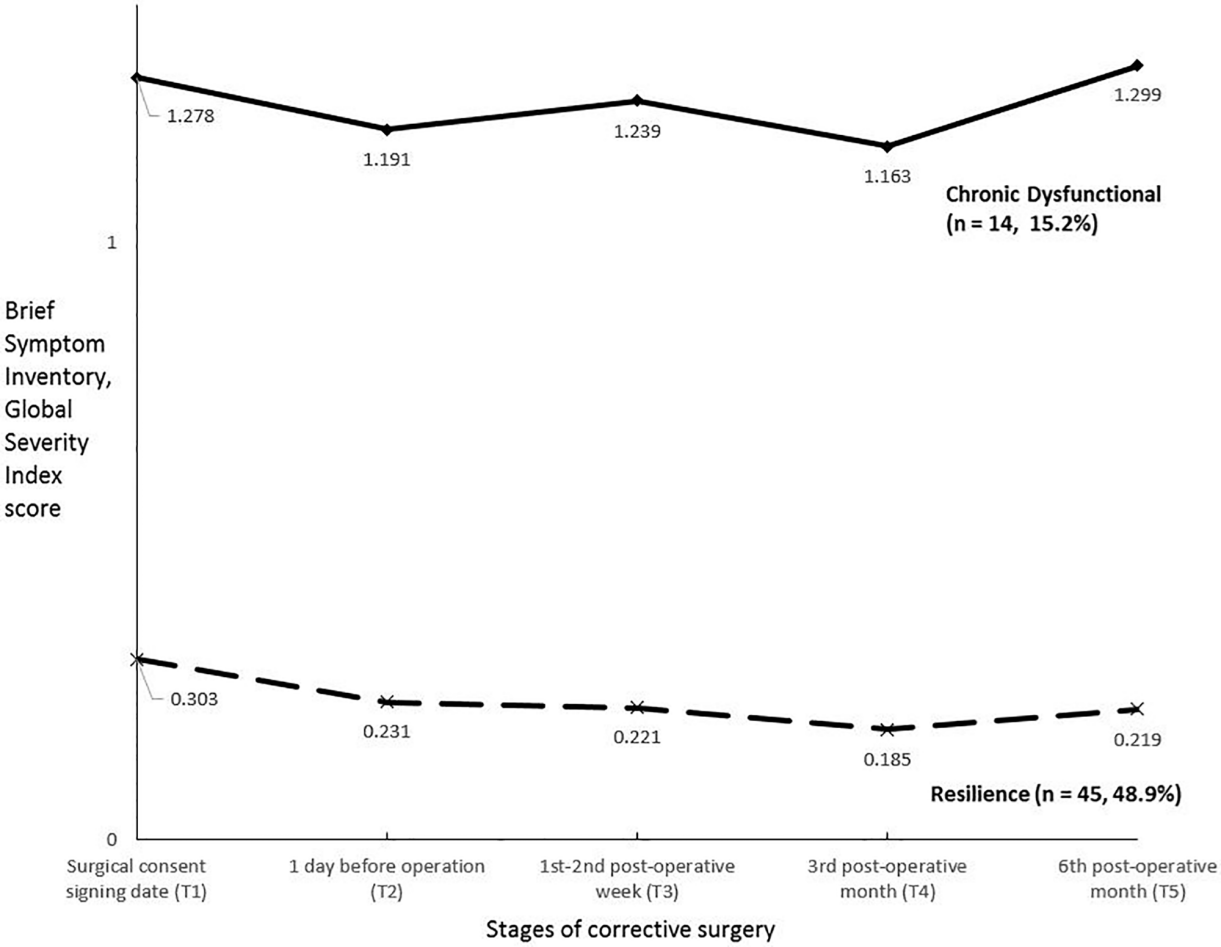
Mean brief symptom inventory global severity index score by prototypical outcome trajectories (n = 92). **Note**: 33 (35.9%) patients did not display meaningful trajectory patterns according to the latent class growth analysis (conditional model) results. These patients were excluded in the above figure.

We used the BSI GSI T-score to classify each subject in Trajectories I and II in each time point as a case (with a T score ≥ 63) or a non-case (with a T score < 63) [20]. All patients in Trajectory I were classified as non-case at all time points. In other words, their psychological distresses were within normal range throughout the surgery process according to the normative data of the BSI. This trajectory was therefore labelled as Resilience trajectory. The opposite was obtained for patients in trajectory II: the distress levels of patients in this trajectory were above the BSI threshold for clinical disorder in all five time points. We labelled this trajectory as Chronic Dysfunction as a result. Fig 2 shows the GSI scores of these two trajectory patterns in different time points following of the corrective surgery.

### Hope and optimism on outcome trajectories of psychological distress

The resilience trajectory (BSI non-case at all time-points) and the chronic dysfunctional trajectory (BSI case in all time-points) are the most reliable indicators on psychological functioning. These two groups were selected to examine whether hope and optimism of the patients would affect the patterns of outcome trajectory.

#### Independent samples t-test

Independent samples t-test was conducted to examine whether the resilience and chronic dysfunctional groups exhibited significantly different hope and optimism levels in each time-points. Results revealed that the resilience group reported higher hope and optimism levels than the chronic dysfunctional group in each time-point. (Table 4)

**Table 4:** Comparison of hope and optimism between the resilient and chronic dysfunctional groups.

	Resilience		Chronic Dysfunctional		t-value
	Mean	SD	Mean	SD	
Surgical Consent Signing date (T1)					
Hope	48.93	7.11	43.43	7.97	2.46*
Optimism	21.18	4.23	16.64	3.18	3.69**
1 day before operation (T2)					
Hope	48.73	6.70	42.15	6.24	3.16**
Optimism	21.62	3.87	17.92	3.62	3.08**
1^st^ -2^nd^ post-operative week (T3)					
Hope	47.96	8.80	41.71	7.90	2.37*
Optimism	21.98	3.62	17.50	3.76	4.01**
3^rd^ post-operative month (T4)					
Hope	48.89	7.09	41.29	7.53	3.45**
Optimism	21.49	3.88	17.50	2.88	3.55**
6^th^ post-operative month (T5)					
Hope	50.18	7.66	38.14	6.81	5.26**
Optimism	21.62	3.86	16.43	3.01	4.61**

Independent-samples t-test; n = 59.

* statistically significant.

** highly statistically significant.

#### Logistic regression

Logistic regression was performed to assess the relative importance of hope and optimism on the likelihood that respondents would be classified as the resilience or non-resilience group. The two variables could predict memberships of outcome trajectory (resilience versus chronic dysfunctional) significantly better than the null model, χ^2^(2) = 13.038, p < .01. These two variables together could correctly predict about 80% of subjects’ membership. As shown in Table 5, optimism score at T1 was statistically significant in predicting the likelihood of the subjects being in the resilient or non-resilient group (B = .276, p < .05). The T1 optimism score recorded an odds ratio of 1.317. This indicated that the odds to be in the resilience group increased by 31.7% by an increase of 1 point in the optimism score. On the other hand, hope was not a significant predictor when optimism was taken into consideration (B = .025, ns). A one-point increase in hope score contributed to about 2.5% increase in likelihood that the subject would become a member of the resilience group.

**Table 5:** Logistic regression predicting likelihood of being in the resilience and chronic dysfunctional outcome trajectory group.

	B	S.E.	Wald’s χ^2^	*df*	*p*	Exp(B) (Odds Ratio)	95.0% C.I. for Odds Ratio	
							Lower	Upper
T1 Hope	.025	.052	.225	1	.635	1.025	.926	1.134
T1 Optimism	.276	.114	5.888	1	.015	1.317	1.054	1.646

## Discussion

### Trajectory pattern of psychological distress

One of the most important contributions of the present study is to establish the outcome trajectory pattern of patients receiving correction surgery for dentofacial deformities. None of the previous studies have investigated this clinical phenomenon before. This study provides a feasible protocol to investigate outcome trajectories among other dental patients in future studies. The most important finding of the present study is that about 15% of patients undergoing dentofacial corrective surgery would exhibit chronic distress during the post-operative period. Appropriate intervention should be implemented to enhance adjustment. Previously, a hope-based intervention programme has been developed to facilitate adjustment of individuals receiving genetic colorectal cancer screening [28]. Similar programmes can be developed for patients undergoing corrective surgery in the future. It is important also to note that, in consistent with previous findings in Hong Kong and elsewhere, most of the patients are resilient–nearly half (48.9%) of the patients would exhibit a resilience outcome trajectory. Proper screening procedures should be developed in future to identify the resilience and non-resilience patients. The Brief Symptom Inventory [20] is a valid and reliable tool for psychological distress screening. Nevertheless, it has 53 items and definitely needs compliance to complete. A shorter 18-item Brief Symptom Inventory (BSI-18) [29] is available but its psychometric properties among dental patients have not been investigated before. We are examining the psychometric properties of the BSI-18 among dental patients and hope to report the results later.

Table 2 shows that the mean BSI GSI score was high at T1 and this reflected that the patients had the highest psychological distress level at that time. Patients planned for jaw correction surgery usually had a joint meeting with the oral and maxillofacial surgeons and orthodontist at T1. During the meeting, they will be informed about the finalised surgical plan for the correction of dentofacial deformities. After that, the surgeon in charge will explain the surgical plan, procedures, risks, potential complications and post-operative hospital care management in detail and have the surgical consent signed by the patients. The stress can be caused by the possibility that they were not yet psychologically well prepared when the surgical details were explained.

The average time lapsed from T1 to T2 is about two to three months. During this period, the patient probably had psychologically prepared or adjusted to face the potential risks, uncertainties ahead and so the BSI GSI score measured at T2 was significantly lower when compared to that at T1. At the early post-operative period (one to two weeks post-operatively), the patients might have increased psychological distress or worries due to post-operative pain, swelling, numbness, temporary inability to open mouth, or other post-operative surgical complications, and might not have adapted to the changes of the new facial appearance yet. This was reflected by the slightly elevated trend of the BSI GSI score at T3, which concurs with the findings from other studies that patients who suffered from surgical complications had significantly worse post-operative psychosocial outcomes even after controlling for preoperative psychosocial outcomes, clinical and demographic factors [30].

At the late post-operative period (T4, third post-operative month), most of the facial swelling would have subsided and wound healing should be completed. The patients suffered less pain or discomfort and they could gradually resume normal diet, mouth opening and social life. They gradually adapted to their new appearances. Hence, the psychological distress level was reduced comparing to that at early post-operative period, as reflected in the BSI GSI score.

### Psychological predictors of trajectory pattern

The present study shows that non-resilient patients, when compared to resilient patients, tended to have lower pre-operative hope and optimism levels (Table 4). Increasing hope and optimism levels of the patients would help to increase resilience of the patients. Strategies to help patients set and achieve realistic goals, as well as maintaining a positive expectation about the surgery would enhance resilience. The logistic regression analysis (Table 5) suggests that optimism is a more important factor than hope in affecting resilience. In other words, surgeons’ attitude to cultivate a good rapport with their patients in order to maintain a positive outlook about the surgery (i.e. increase optimism) is possibly the most important factor in enhancing resilience from the present study. Existing psychological programmes to increase optimism in other populations may be adapted to dental patients [31].

### Limitations

There are several limitations related to this study. Firstly, other trajectory patterns such as the recovery and delayed reaction patterns (Fig 1) were not identified in the present study, and about 35% of the patients could not be classified into any trajectory patterns. A larger sample size might enable us to identify other trajectory patterns. Secondly, previous studies have suggested that the severity of the stressful event is an important factor affecting the prevalence of outcome trajectories. Individuals exposed to extremely stressful events exhibited a higher prevalence of psychopathology and a lower prevalence of resilience in comparison with those exposed to low-stress levels [3]. The high prevalence of resilience trajectories and the low prevalence of chronic dysfunction trajectories in our study suggest that jaw correction surgery for dentofacial deformities induces a mild to moderate level of stress among patients. Our results may not be generalizable to dental patients undergoing severe intrusive operations. Thirdly, the whole set of questionnaires comprises 71 questions which required 10 to 15 minutes to complete. About 30% of the recruited subjects failed to complete all the questionnaires at all time points. Among all, BSI-53 is the most lengthy one which contains 53 questions. A shortened validated version of BSI may be helpful in shortening the completion time and may thus improve the compliance in completing the questionnaires. Finally, this study focused on the longitudinal psychological changes of patients with dentofacial deformity after jaw correction surgery up to post-operative six months. Data for a longer post-operative periods e.g. up to post-operative one year will be helpful to generate a comparison regarding the longitudinal post-operative psychological changes at early post-operative period to late post-operative period.

## Conclusion

Nearly half of patients with dentofacial deformity underwent jaw correction surgery exhibited a resilience trajectory patterns up to 6 months post-operatively. About 15% of them exhibited clinical level of psychological distress throughout the early post-operative period. Optimism and to a lesser extent hope at the pre-surgical phase are related to better adjustment. Proper prophylactic psychological interventions to increase realistic optimism and hope related to the surgery may reduce post-operative chronic dysfunctional distress.

## Remarks

The above manuscript was in reference to first author’s dissertations submitted to the Faculty of Dentistry, The University of Hong Kong as “Prospective study of changes in psychosocial characteristics of patients with dentofacial deformities after corrective surgery” for the degree of Master of Dental Surgery and as “Changes in psychosocial characteristics of patients with dentofacial deformities after corrective surgery: 1-year prospective study” for advanced diploma of Oral and Maxillofacial Surgery in 2013 and 2015 respectively.
